# Neimark-Sacker bifurcation of a two-dimensional discrete-time predator-prey model

**DOI:** 10.1186/s40064-015-1618-y

**Published:** 2016-02-18

**Authors:** A. Q. Khan

**Affiliations:** Department of Mathematics, University of Azad Jammu and Kashmir, Muzaffarabad, 13100 Pakistan; Department of Mathematics, Shanghai Jiao Tong University, Shanghai, 200240 China

**Keywords:** Predator-prey model, Stability, Neimark-Sacker bifurcation, Bifurcation theory, 37D45, 37G35, 39A30, 39A33

## Abstract

In this paper, we study the dynamics and bifurcation of a two-dimensional discrete-time predator-prey model in the closed first quadrant $\mathbb {R}_+^2$. The existence and local stability of the unique positive equilibrium of the model are analyzed algebraically. It is shown that the model can undergo a Neimark-Sacker bifurcation in a small neighborhood of the unique positive equilibrium and an invariant circle will appear. Some numerical simulations are presented to illustrate our theocratical results and numerically it is shown that the unique positive equilibrium of the system is globally asymptotically stable.

## Background

As is well known, in the theory of population dynamical models there are two kinds of mathematical models: the continuous-time models described by differential equations, and the discrete time models described by difference equations. In recent years more and more attention is being paid to discrete time population models. The reasons are as follows: First, the discrete time models are more appropriate than the continuous time models when populations have non-overlapping generations, or the number of population is small. Second, we can get more accurate numerical simulations results from discrete time models. Moreover, the numerical simulations of continuous-time models are obtained by discretizing the models. At last, the discrete-time models have rich dynamical behaviors as compared to continuous time models. Predator-prey models have already received much attention during last few years. For example, the stability, permanence and the existence of periodic solutions of the predator-prey models are studied in Fazly (2007), Hu and Zhang (2008), Liu (2010), Xia et al. (2007) and Yang and Li (2009). Study of discrete dynamical behavior of systems is usually focus on boundedness and persistence, existence and uniqueness of equilibria, periodicity, and there local and global stability (see for example, Khan and Qureshi 2014a, b, 2015a, b, c; Kalabuŝić et al. 2009; Khan 2014; Ibrahim and El-Moneam 2015; Kalabuŝić et al. 2011; Elsayed and Ibrahim 2015a, b; Garić-Demirović et al. 2009; Qureshi and Khan 2015; Kalabuŝić et al. 2011; Ibrahim 2014; Ibrahim and Touafek 2014) but there are many articles that discuss the dynamical behavior of discrete-time models for exploring the possibility of bifurcation and chaos phenomena (Hu et al. 2011; Sen et al. 2012; Chen and Changming 2008; Gakkhar and Singh 2012; Jing and Yang 2006; Zhang et al. 2010; Smith 1968).

We consider the following discrete predator-prey model described by difference equations which was proposed by Smith et al. (1968):

1 $$\begin{aligned} x_{n+1} = \alpha x_n(1-x_n)- x_ny_n,\quad y_{n+1} = \frac{1 }{\beta }x_ny_n, \end{aligned}$$

where $x_n$ and $y_n$ denotes the numbers of prey and predator respectively. Moreover the parameters $\alpha ,\ \beta$ and the initial conditions $x_0,\ y_0$ are positive real numbers.

The organization of the paper is as follows: In Sect. “Existence of equilibria and local stability”, we discuss the existence and local stability of equilibria for system (1) in $\mathbb {R}_+^2$. This also include the specific parametric condition for the existence of Neimark-Sacker bifurcation of the system (1). In Sect. “Neimark-Sacker bifurcation”, we study the Neimark-Sacker bifurcation by choosing $\alpha$ as a bifurcation parameter. In Sect. “Numerical simulations”, numerical simulations are presented to verify theocratical discussion. Finally a brief conclusion is given in Sect. “Conclusion”.

## Existence of equilibria and local stability

In this section, we will study the existence and stability of equilibria of system (1) in the close first quadrant $\mathbb {R}^2_{+}$. So, we can summarized the results about the existence of equilibria of system (1) as follows:

### **Lemma 2.1**

(i) *System* (1)* has a unique equilibrium O(0, 0) if *$\alpha <\frac{1}{1-\beta }$* and *$\beta <1$;
(ii) *System* (1)* has two equilibriaO(0, 0)and *$A\left( \beta ,\alpha (1-\beta )-1\right)$* if*$\alpha >\frac{1}{1-\beta }$* and*$\beta <1$.* More precisely, system *(1)* has a unique positive equilibrium*$A\left( \beta ,\alpha (1-\beta )-1\right)$* if*$\alpha >\frac{1}{1-\beta }$* and*$\beta <1$.

Now we will study the dynamics of system (1) about these equilibria. The Jacobian matrix of linearized system of (1) about the equilibrium (*x*, *y*) is

2 $$\begin{aligned} J=\left( \begin{array}{cc} \alpha -2\alpha x-y&{} -x\\ \frac{1}{\beta }y&{} \frac{1}{\beta }x \end{array} \right) . \end{aligned}$$

The characteristic equation of the Jacobian matrix *J* of linearized system of (1) about the unique positive equilibrium $A\left( \beta ,\alpha (1-\beta )-1\right)$ is given by

3 $$\begin{aligned} \lambda ^2+p\lambda +q=0, \end{aligned}$$

where

$$\begin{aligned} p&=2-\alpha \beta ,\quad q&=\alpha -2\alpha \beta . \end{aligned}$$

Moreover the eigenvalues of the Jacobian matrix of linearized system of (1) about the unique positive equilibrium $A\left( \beta ,\alpha (1-\beta )-1\right)$ is given by

$$\begin{aligned} \lambda _{1,2}=\frac{-p\pm \sqrt{\Delta }}{2} \end{aligned}$$

where

$$\begin{aligned} \Delta\;=\;&  p^2-4q,\\\;=\;& (\alpha \beta +2)^2-4\alpha . \end{aligned}$$

Now we will state the topological classification of these equilibria as follows:

### **Lemma 2.2**

(i) *For the equilibrium point O*(0, 0),* following topological classification holds:*
  1. *O(0, 0) is a sink if *$\alpha <1$;
  2. *O*(*0, 0) is a saddle if*$\alpha >1$;
  3. *O(0, 0) is non-hyperbolic if *$\alpha =1$.

### **Lemma 2.3**

*For the unique positive equilibrium*$A\left( \beta ,\alpha (1-\beta )-1\right)$* of system *(1),* following topological classification holds:*

(i) $A\left( \beta ,\alpha (1-\beta )-1\right)$* is a sink if one of the following parametric conditions holds:*
  1. $$\Delta \ge 0$$* and *$$0<\alpha <\frac{1}{1-\beta }$$;
  2. $$\Delta <0$$* and *$$0<\alpha <\frac{1}{1-2\beta }$$ ;
(ii) $A\left( \beta ,\alpha (1-\beta )-1\right)$* is a source if one of the following parametric conditions holds:*
  1. $$\Delta \ge 0$$* and *$$\alpha >\frac{1}{1-\beta }$$;
  2. $$\Delta <0$$* and *$$\alpha >\frac{1}{1-2\beta }$$;
(iii) $A\left( \beta ,\alpha (1-\beta )-1\right)$* is non-hyperbolic if one of the following parametric conditions holds:*
  1. $$\Delta \ge 0$$* and *$$\alpha =\frac{1}{1-\beta }$$;
  2. $$\Delta <0$$* and*$$\alpha =\frac{1}{1-2\beta }$$;

From Lemmas 2.2 and 2.3, we summarize the local dynamics of system (1) as follows:

### **Theorem 2.4**

(i) *If *$\alpha <\frac{1}{1-\beta }$* and*$\beta <1$,* then system *(1)* has a unique equilibriumO*(*0, 0*),* which is locally asymptotically stable;*
(ii) *If *$\alpha >\frac{1}{1-\beta }$* and*$\beta <1$,* then system* (1)* has two equilibriaO*(*0, 0*)* and *$A\left( \beta ,\alpha (1-\beta )-1\right)$,* in which*$A\left( \beta ,\alpha (1-\beta )-1\right)$ i*s locally asymptotically stable*.

In the following section, we will study the Neimark-Sacker bifurcation about the unique positive equilibrium $A\left( \beta ,\alpha (1-\beta )-1\right)$ by using bifurcation theory (Guckenheimer and Holmes 1983; Kuznetson 2004).

## Neimark-Sacker bifurcation

From Lemma 2.3, it is established that $A\left( \beta ,\alpha (1-\beta )-1\right)$ is non-hyperbolic when $\alpha =\frac{1}{1-2\beta }$. Henceforth, we choose $\alpha$ as a bifurcation parameter to study Neimark-Sacker bifurcation of system (1) in the small neighborhood of $A\left( \beta ,\alpha (1-\beta )-1\right)$. For simplicity, we denote the parameters satisfying non-hyperbolic condition by

$$\begin{aligned} H_A=\left\{ (\alpha ,\beta ){:}\Delta <0,\;\alpha =\frac{1}{1-2\beta },\; \beta <\frac{1}{2}, \; \alpha ,\beta >0 \right\} . \end{aligned}$$

Consider system (1) with arbitrary parameters $(\alpha _1,\beta _1)\in H_A$, which is described as follows:

4 $$\begin{aligned} x_{n+1} = \alpha _1 x_n(1-x_n)- x_ny_n,\quad \ y_{n+1} = \frac{1 }{\beta _1}x_ny_n. \end{aligned}$$

It is clear that if $\alpha _1>\frac{1}{1-\beta _1}\ and \ \beta _1<1$, then $A\left( \beta _1,\alpha _1(1-\beta _1)-1\right)$ has a unique positive equilibrium of system (4). Given a perturbation of model (4) as follows:

5 $$\begin{aligned} x_{n+1} = (\alpha _1+\alpha ^*) x_n(1-x_n)- x_ny_n,\quad \ y_{n+1} = \frac{1 }{\beta _1}x_ny_n., \end{aligned}$$

where $|\alpha ^*|\ll 1$, which is small parameters.

The characteristic equation of the Jacobian matrix of linearized system of (5) about the unique positive equilibrium $A\left( \beta _1,\alpha _1(1-\beta _1)-1\right)$ is given by

$$\begin{aligned} \lambda ^2-p(\alpha ^*)\lambda +q(\alpha ^*)=0, \end{aligned}$$

where

$$\begin{aligned} p(\alpha ^*)= & {} 2-(\alpha _1+\alpha ^*)\beta _1,\quad \ q(\alpha ^*)=(\alpha _1+\alpha ^*)-2(\alpha _1+\alpha ^*)\beta _1. \end{aligned}$$

Moreover when $\alpha ^*$ varies in a small neighborhood of 0, the roots of the characteristic equation are

$$\begin{aligned} \lambda _{1,2}\;=\;&  \frac{-p(\alpha ^*)\mp \iota \sqrt{4q(\alpha ^*)-p^2(\alpha ^*)}}{2},\\\;=\; &  \frac{(\alpha _1+\alpha ^*)\beta _1-2\mp \iota \sqrt{4(\alpha _1+\alpha ^*)-((\alpha _1+\alpha ^*)\beta _1+2)^2}}{2}, \end{aligned}$$

and there we have

$$\begin{aligned} |\lambda _{1,2}|=(q(\alpha ^*))^{\frac{1}{2}}, \quad \ \frac{d|\lambda _{1,2}|}{d \alpha ^*}|_{\alpha ^*=0}=\frac{1-2\beta _1}{2}>0. \end{aligned}$$

Further calculation shows that $\lambda _{1,2}^k\ne 1$ for $\alpha _1=\frac{1}{1-2\beta _1}$ and $k=1,2,3,4$. Now, let $u_n=x_n-\beta _1, v_n=y_n-\alpha _1(1-\beta _1)+1$, then we transform the equilibrium $A\left( \beta _1,\alpha _1(1-\beta _1)-1\right)$ of system (5) into the origin. By calculating we obtain

6 $$\begin{aligned} u_{n+1}&\;=\;(\alpha _1+\alpha ^*) (u_n+\beta _1)(1-u_n-\beta _1)- (u_n+\beta _1)(v_n+\alpha _1(1-\beta _1)-1)-\beta _1, \nonumber \\ v_{n+1}\;&=\;\frac{1}{\beta _1}(u_n+\beta _1)(v_n+\alpha _1(1-\beta _1)-1)-\alpha _1(1-\beta _1)+1. \end{aligned}$$

In the following, we study the normal form of system (6) when $\alpha ^*=0$. Expanding (6) as a Taylor series at $(u_n,v_n)=(0,0)$, we get

7 $$\begin{aligned} u_{n+1} &= {} a_{11}u_n+a_{12}v_n+a_{13}u_n^2+a_{14}u_nv_n, \nonumber \\ v_{n+1} &= {} a_{21}u_n+a_{22}v_n+a_{23}u_nv_n, \end{aligned}$$

where

$$\begin{aligned} a_{11}\;=\;& {} 1-\alpha _1\beta _1, \quad \ a_{12}=-\beta _1,\quad \ a_{13}=-\alpha _1, \quad \ a_{14}=-1,\\ a_{21}\;=\; & {} \frac{\alpha _1(1-\beta _1)-1}{\beta _1},\quad \ a_{22}=1,\quad \ a_{23}=\frac{1}{\beta _1}. \end{aligned}$$

Now, let

$$\begin{aligned} \eta =\frac{\alpha _1\beta _1-2}{2},\quad \ \zeta =\frac{1}{2}\sqrt{4\alpha _1-(\alpha _1\beta _1+2)^2}, \end{aligned}$$

and

$$\begin{aligned} T=\left( \begin{array}{cc} a_{12}&{}0 \\ \eta -a_{11}&{}-\zeta \\ \end{array} \right) , \end{aligned}$$

then *T* is invertible. Using translation

$$\begin{aligned} \left( \begin{array}{c} u_n \\ v_n \\ \end{array} \right) =\left( \begin{array}{cc} a_{12}&{}0 \\ \eta -a_{11}&{}-\zeta \\ \end{array} \right) \left( \begin{array}{c} {X_n} \\ {Y_n} \\ \end{array} \right) , \end{aligned}$$

then system (7) becomes of the following form:

8 $$\begin{aligned} X_{n+1}&=\eta X_n-\zeta Y_n+ \bar{F}(X_n,Y_n), \nonumber \\ Y_{n+1}&=\zeta X_n+\eta Y_n+ \bar{G}(X_n,Y_n), \end{aligned}$$

where

9 $$\begin{aligned} \bar{F}(X_n,Y_n)&=c_{11}X_n^2+c_{12}X_nY_n, \nonumber \\ \bar{G}(X_n,Y_n)&=c_{21}X_n^2+c_{22}X_nY_n, \end{aligned}$$

and

$$\begin{aligned} c_{11}\;=\; &  a_{12}a_{13}+(\eta -a_{11})a_{14},\ c_{12}=-\zeta ,\\ c_{21}\;=\; &  \frac{1}{\zeta }\left[ (\eta -a_{11})a_{12}a_{13}+((\eta -a_{11})a_{14}-a_{23})(\eta -a_{11})a_{12}\right] ,\\ c_{22}\;=\; &  \left[ a_{23}-\frac{(\eta -a_{11})a_{14}}{a_{12}}\right] a_{12}. \end{aligned}$$

Furthermore,

$$\begin{aligned} \bar{F}_{X_nX_n}\big |_{(0,0)}\;=\; & 2c_{11},\ \bar{F}_{X_nY_n}\big |_{(0,0)}=c_{12},\ \bar{F}_{Y_nY_n}\big |_{(0,0)}=0,\\ \bar{F}_{X_nX_nX_n}\big |_{(0,0)}\;= \;& \bar{F}_{X_nX_nY_n}\big |_{(0,0)}=\bar{F}_{X_nY_nY_n}\big |_{(0,0)}=\bar{F}_{Y_nY_nY_n}\big |_{(0,0)}=0, \end{aligned}$$

and

$$\begin{aligned} \bar{G}_{X_nX_n}|_{(0,0)}\;=\; & 2c_{21},\ \bar{G}_{X_nY_n}|_{(0,0)}=c_{22},\ \bar{G}_{Y_nY_n}|_{(0,0)}=0,\\ \bar{G}_{X_nX_nX_n}|_{(0,0)}\;= \;& \bar{G}_{X_nX_nY_n}|_{(0,0)}=\bar{G}_{X_nY_nY_n}|_{(0,0)}=\bar{G}_{Y_nY_nY_n}|_{(0,0)}=0. \end{aligned}$$

In order to guarantee the Neimark-Sacker bifurcation for (8), we require that the following discriminatory quantity is not zero (Guckenheimer and Holmes 1983):

10 $$\begin{aligned} \Omega =-Re\left[ \frac{(1-2\bar{\lambda })\bar{\lambda }^2}{1-\lambda }\xi _{11}\xi _{20}\right] -\frac{1}{2}\Vert \xi _{11}\Vert ^2-\Vert \xi _{02}\Vert ^2+Re(\bar{\lambda }\xi _{21}), \end{aligned}$$

where

11 $$ \begin{aligned}   \xi _{{02}}  &  = \frac{1}{8}\left[ {\bar{F}_{{X_{n} X_{n} }}  - \bar{F}_{{Y_{n} Y_{n} }}  + 2\bar{G}_{{X_{n} Y_{n} }}  + \iota \left( {\bar{G}_{{X_{n} X_{n} }}  - \bar{G}_{{Y_{n} Y_{n} }}  + 2\bar{F}_{{X_{n} Y_{n} }} } \right)} \right]|_{{(0,0)}} , \\    \xi _{{11}}  &  = \frac{1}{4}\left[ {\bar{F}_{{X_{n} X_{n} }}  + \bar{F}_{{Y_{n} Y_{n} }}  + \iota \left( {\bar{G}_{{X_{n} X_{n} }}  + \bar{G}_{{Y_{n} Y_{n} }} } \right)} \right]|_{{(0,0)}} , \\    \xi _{{20}}  &  = \frac{1}{8}\left[ {\bar{F}_{{X_{n} X_{n} }}  - \bar{F}_{{Y_{n} Y_{n} }}  + 2\bar{G}_{{X_{n} Y_{n} }}  + \iota \left( {\bar{G}_{{X_{n} X_{n} }}  - \bar{G}_{{Y_{n} Y_{n} }}  - 2\bar{F}_{{X_{n} Y_{n} }} } \right)} \right]|_{{(0,0)}} , \\    \xi _{{21}}  &  = \frac{1}{{16}}\left\{ {\bar{F}_{{X_{n} X_{n} X_{n} }}  + \bar{F}_{{X_{n} Y_{n} Y_{n} }}  + \bar{G}_{{X_{n} X_{n} Y_{n} }}  + \bar{G}_{{Y_{n} Y_{n} Y_{n} }} } \right.\; \\ & \quad \left.  {+ \iota \left( {\bar{G}_{{X_{n} X_{n} X_{n} }}  + \bar{G}_{{X_{n} Y_{n} Y_{n} }}  - \bar{F}_{{X_{n} X_{n} Y_{n} }}  - \bar{F}_{{Y_{n} Y_{n} Y_{n} }} } \right)} \right\}|_{{(0,0)}} . \\  \end{aligned}  $$

After calculating, we get

12 $$\begin{aligned} \xi _{02}&=\frac{1}{4}\left[ c_{11}+c_{22}+\iota (c_{21}+c_{12})\right] ,\nonumber \\ \xi _{11}&=\frac{1}{2}\left[ c_{11}+\iota c_{21}\right] ,\nonumber \\ \xi _{20}&=\frac{1}{4}\left[ c_{11}+c_{22}+\iota (c_{21}-c_{12})\right] ,\nonumber \\ \xi _{21}&= 0, \end{aligned}$$

Analyzing the above and the Neimark- Sacker bifurcation conditions discussed in Guckenheimer and Holmes (1983), we write the theorem as follows:

### **Theorem 3.1**

*If the condition *(10)* holds*, *i*.*e*., $\Omega \not =0$* and the parameter*$\alpha$* alters in the limited region of the point (0, 0), then the system* (4)* passes through a Neimark- Sacker bifurcation at the unique positive equilibrium*$A\left( \beta _1,\alpha _1(1-\beta _1)-1\right)$.* Moreover, if *$\Omega <0$ (*respectively*$\Omega >0$),* then an attracting (respectively repelling) invariant closed curve bifurcates from the equilibrium*$A\left( \beta ,\alpha (1-\beta )-1\right)$* for*$\alpha >0$ (*respectively*$\alpha <0$).

## Numerical simulations

**Fig. 1:**
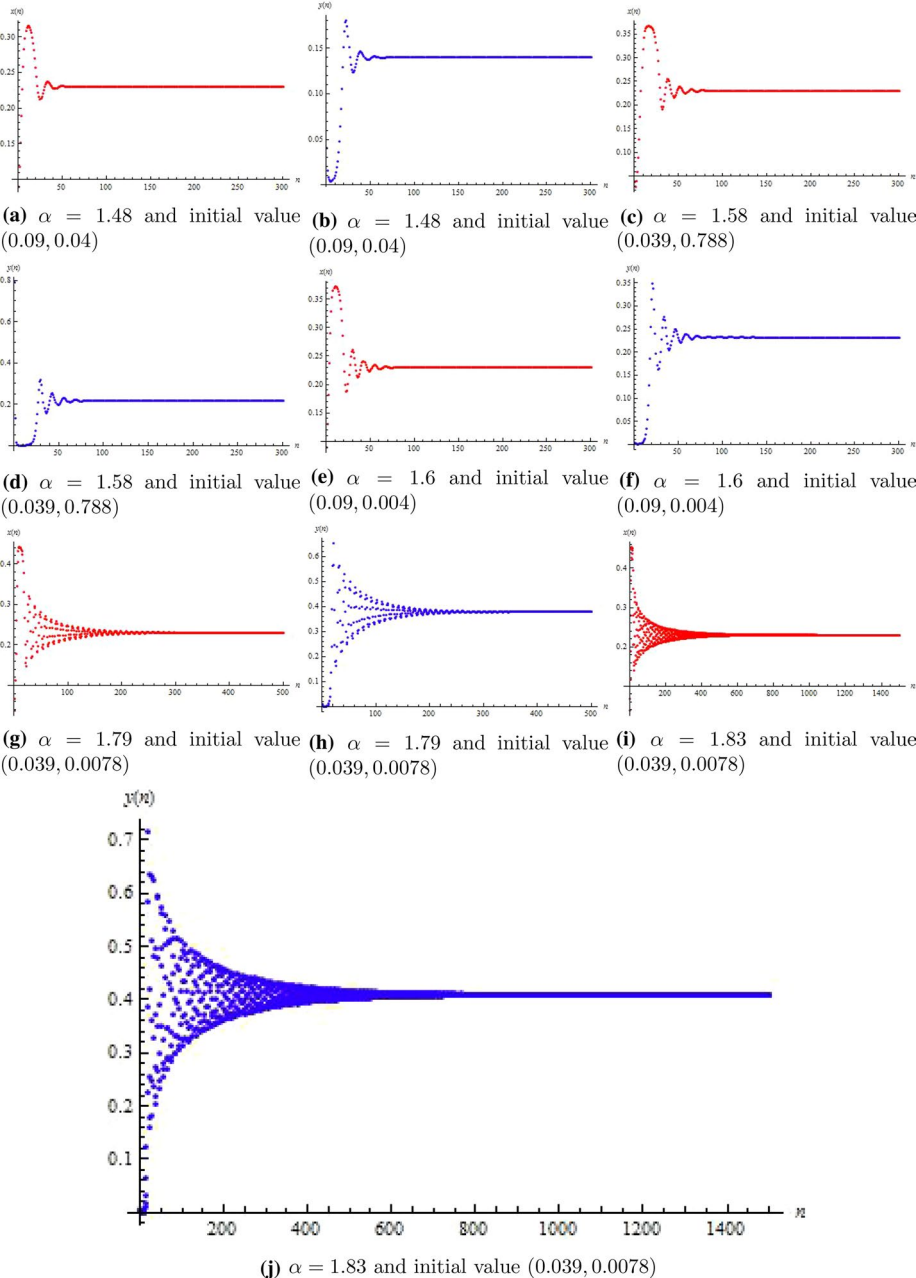
Phase portraits of system (1)

**Fig. 2:**
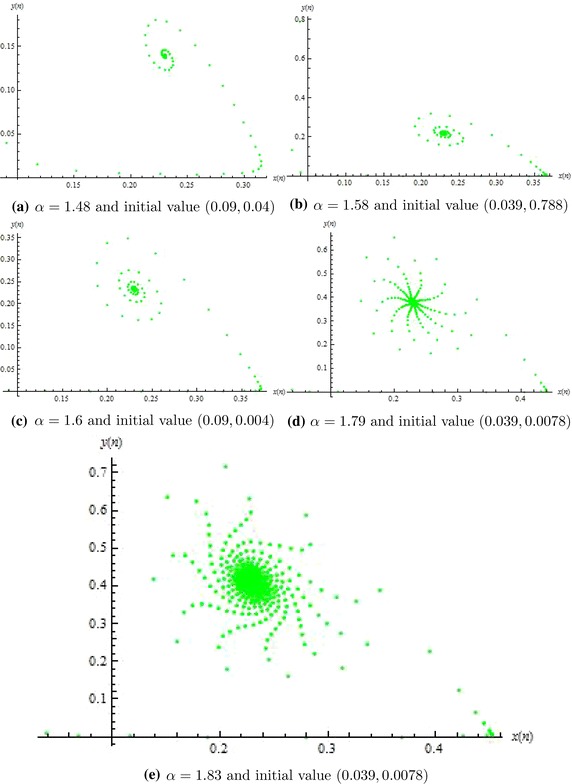
Phase portraits of system (1)

**Fig. 3:**
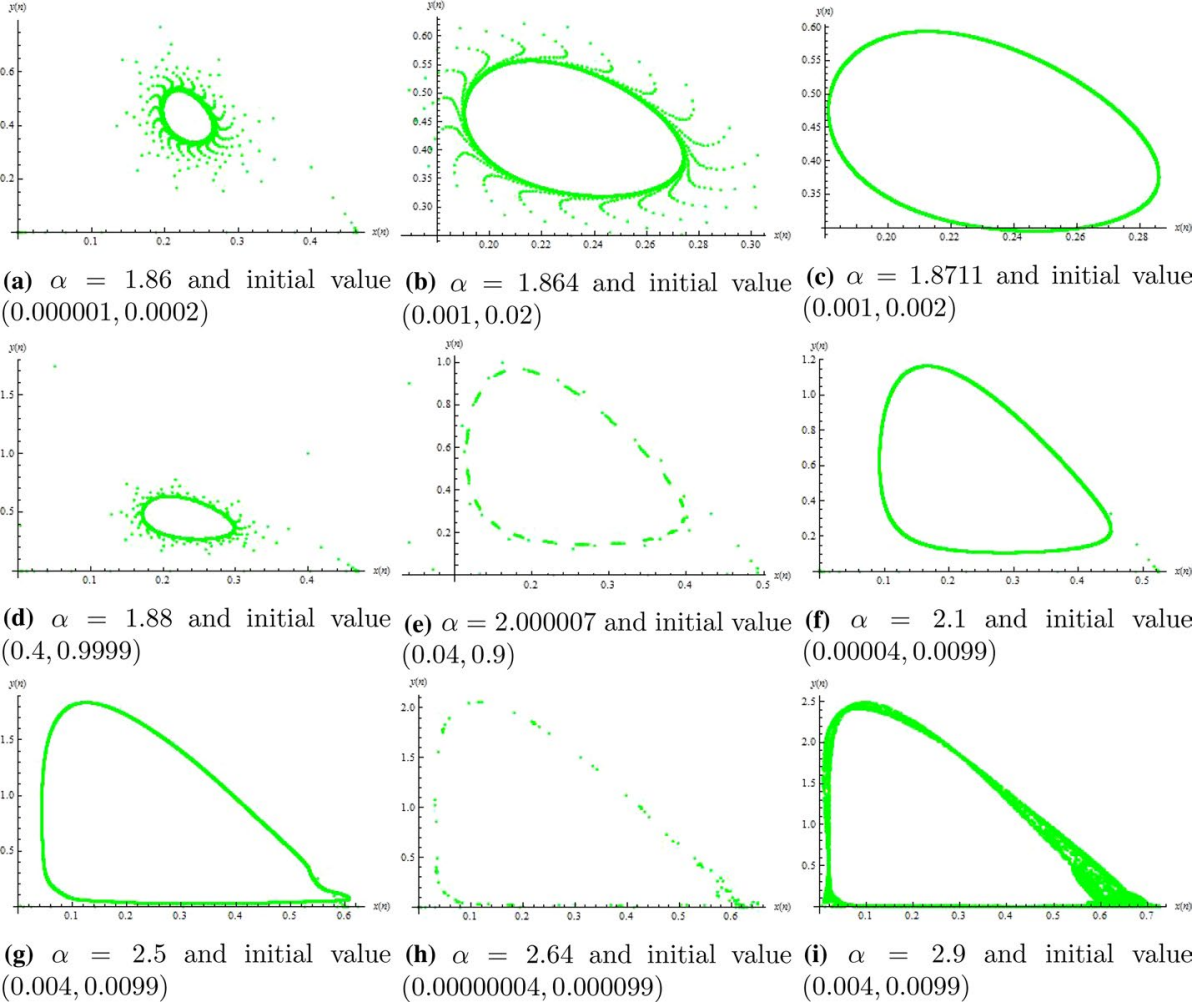
Phase portraits of system (1)

In this section, we will give some numerical simulations for the system (1) to support our theoretical results. If we choose $\beta =0.23$, then from non-hyperbolic condition (*iii*.2) of Lemma 2.3, the value of bifurcation parameter is $\alpha =1.85185$. In theoretical point of view, the unique positive equilibrium is stable if $\alpha <1.85185$, loss its stability at $\alpha =1.85185$ and an attracting invariant close curves appear from the positive equilibrium when $\alpha >1.85185$. From subfigures a and b of Fig. 1 it is clear that if $\alpha =1.48<1.85185$, then unique positive equilibrium is locally stable and corresponding to Fig. 1a, b one can easily seen from Fig. 2a that it is an attractor. So, Fig. 1 shows the local stability of system (1) whereas Fig. 2 shows that the unique positive equilibrium of system (1) is globally asymptotically stable. Figure 3 shows that for different choices of parameters when $\alpha >1.85185$, then unique positive equilibrium is unstable and meanwhile an attracting invariant closed curve bifurcates from the positive equilibrium, as in Fig. 3a–i.

## Conclusion

This work is related to stability and bifurcation analysis of a discrete predator-pray model. We proved that system (1) have two equilibria namely (0, 0) and $A\left( \beta ,\alpha (1-\beta )-1\right)$. Moreover, simple algebra shows that if $\alpha >\frac{1}{1-\beta },\ \beta <1$ then system (1) has unique positive equilibrium $A\left( \beta ,\alpha (1-\beta )-1\right)$. The method of linearization is used to prove the local asymptotic stability of equilibria. Linear stability analysis shows that *O*(0, 0) is a sink if $\alpha <1$, saddle if $\alpha >1$, and non-hyperbolic if $\alpha =1$. For the unique positive equilibrium $A\left( \beta ,\alpha (1-\beta )-1\right)$, we have different topological types for possible parameters and proved that it is locally asymptotically stable and under the condition $\alpha =\frac{1}{1-2\beta }$ the eigenvalues of the Jacobian matrix are a pair of complex conjugate with modulus one. This means that there exist a Neimark-Saker bifurcation when the parameters vary in the neighborhood of $H_A$. Then we present the Neimark-Saker bifurcation for the unique positive equilibrium point $A\left( \beta ,\alpha (1-\beta )-1\right)$ of system (1) by choosing $\alpha$ as a bifurcation parameter. We analysis the Neimark-Sacker bifurcation both by theoretical point of view and by numerical simulations. These numerical examples are experimental verifications of theoretical discussions.
